# Inter-individual cognitive variability in children with Asperger's syndrome

**DOI:** 10.3389/fnhum.2014.00575

**Published:** 2014-07-31

**Authors:** Maria Luz Gonzalez-Gadea, Paula Tripicchio, Alexia Rattazzi, Sandra Baez, Julian Marino, Maria Roca, Facundo Manes, Agustin Ibanez

**Affiliations:** ^1^Laboratory of Experimental Psychology and Neuroscience, Institute of Cognitive Neurology, Favaloro UniversityBuenos Aires, Argentina; ^2^National Scientific and Technical Research CouncilBuenos Aires, Argentina; ^3^UDP-INECO Foundation Core on Neuroscience, Diego Portales UniversitySantiago, Chile; ^4^Universidad Catolica ArgentinaBuenos Aires, Argentina; ^5^Facultad de Psicología, Universidad Nacional de CórdobaCordoba, Argentina; ^6^Australian Research Council, Centre of Excellence in Cognition and its DisordersSydney, NSW, Australia; ^7^Universidad Autonoma del CaribeBarranquilla, Colombia

**Keywords:** individual variability, fluid intelligence, theory of mind, executive functions, Asperger's syndrome

## Abstract

Multiple studies have tried to establish the distinctive profile of individuals with Asperger's syndrome (AS). However, recent reports suggest that adults with AS feature heterogeneous cognitive profiles. The present study explores inter-individual variability in children with AS through group comparison and multiple case series analysis. All participants completed an extended battery including measures of fluid and crystallized intelligence, executive functions, theory of mind, and classical neuropsychological tests. Significant group differences were found in theory of mind and other domains related to global information processing. However, the AS group showed high inter-individual variability (both sub- and supra-normal performance) on most cognitive tasks. Furthermore, high fluid intelligence correlated with less general cognitive impairment, high cognitive flexibility, and speed of motor processing. In light of these findings, we propose that children with AS are characterized by a distinct, uneven pattern of cognitive strengths and weaknesses.

## Introduction

Autism spectrum disorders (ASD) are a group of neuro-developmental conditions that compromises social interaction (including verbal and non-verbal communication), and presents restricted, repetitive interests and activities (American Psychiatric Association, 2013). Asperger's syndrome (AS) is a subset of ASD, with an absence of cognitive, developmental or language delay in childhood (Woodbury-Smith and Volkmar, 2009; Durdiakova et al., 2014). Individual differences in AS suggest a heterogeneous neuro-cognitive profile. Cognitive impairments have been observed in executive functions (EFs; Ambery et al., 2006; Hill and Bird, 2006; Semrud-Clikeman et al., 2010), theory of mind (ToM; Baron-Cohen et al., 1999, 2001c; Nagar Shimoni et al., 2012), and global information processing (Happe and Frith, 2006; Bowler et al., 2008; Le Sourn-Bissaoui et al., 2011), whereas some strengths have been observed in abstract problem-solving or fluid intelligence (Hayashi et al., 2008; Chen et al., 2010; Soulieres et al., 2011). However, considerable inconsistencies exist in the literature, as no single explanation has been able to account for all the cognitive strengths and weaknesses of AS.

Executive functioning, planning, and cognitive flexibility are cognitive processes consistently reported as impaired in AS children (Liss et al., 2001; Happe et al., 2006a; Semrud-Clikeman et al., 2010) and adults (Kleinhans et al., 2005; Ambery et al., 2006). However, results have been mixed, as some studies have shown no differences in either children (Kaland et al., 2008b; Van Eylen et al., 2011) or adults (Hill and Bird, 2006; Kenworthy et al., 2008).

Regarding ToM, AS children (Kaland et al., 2002; Le Sourn-Bissaoui et al., 2011) and adults (Baron-Cohen et al., 2001a; Zalla et al., 2009) sometimes fail to infer mental states in themselves or others. Nevertheless, studies in AS adults found preserved performance in the Reading-the-Mind-in-the-Eyes test (RMET, Ponnet et al., 2004; Spek et al., 2010; Baez et al., 2012; Gonzalez-Gadea et al., 2013).

AS individuals also exhibit a reduced ability to process information in context, thus favoring local over global processing. Consequently, AS children (Koyama and Kurita, 2008; Chen et al., 2009; Girardot et al., 2012) and adults (Spek et al., 2008) outperform control groups on tasks that depend on the processing of local features (e.g., embedded figures task and block design). Conversely, they show deficits on tasks that require global processing, such as the Rey Complex Figure Test (RCFT; Kuschner et al., 2009; Le Sourn-Bissaoui et al., 2011). Nevertheless, this picture is complicated by the report of opposite results in both children (Schlooz et al., 2006; Kaland et al., 2007; Manjaly et al., 2007; Chen et al., 2008; de Jonge et al., 2009) and adults (Jolliffe and Baron-Cohen, 1997). In this sense, Bowler et al. (2008) have shown that AS adults tend to organize information in an idiosyncratic way. They fail to learn and recall successive lists of semantically related words and organize recall mainly in terms of the lists' structure rather than through semantic or associative features (Bennetto et al., 1996; Bowler et al., 2008). However, in the study by Smith et al. (2007), AS adults and controls showed no differences in encoding and storing a list of unrelated words. At present, performance of AS children on these tasks remains unknown.

Finally, individuals with AS feature an atypical profile of intelligence, including better verbal than performance IQ (Klin et al., 1995) and enhanced abstract reasoning or fluid intelligence (FI; Hayashi et al., 2008; Morsanyi and Holyoak, 2010; Soulieres et al., 2011). Furthermore, a recent study (Soulieres et al., 2011) suggests that superior FI in AS may imply a common mechanism advantageously applied to solve cognitive tasks.

In sum, the nature of the strengths and weaknesses of children with AS is still a matter of debate. To date, no single theory has been able to account for the core features of the syndrome. Moreover, recent studies on AS (Happe et al., 2006b; Brunsdon and Happe, 2014) suggest high inter-individual cognitive variability, which may reflect an abnormal pattern of neurofunctional specialization in autistic individuals (Pierce et al., 2001; Cherkassky et al., 2006; Gilbert et al., 2009). Indeed, the association between cognitive performance variability and atypical brain organization has been corroborated through novel neuropsychological approaches (Hill and Bird, 2006; Towgood et al., 2009; Pellicano, 2010; Baez et al., 2012; Gonzalez-Gadea et al., 2013).

In particular, a recent methodology called multiple case series analysis (MCSA; Hill and Bird, 2006; Towgood et al., 2009; Baez et al., 2012; Gonzalez-Gadea et al., 2013) has been used to study inter-individual variability in this population. This approach relies on detailed analyses of individual cases to detect the domains in which a single member shows extreme performance. Traditional group-study analysis is not well-suited for individuals with high performance variability because of the averaging artifact (Shallice and Evans, 1978). In other words, heterogeneity is concealed in groups featuring large individual differences. Likewise, in group comparison studies, effect sizes tend to be small, if not altogether omitted from the reports. By exploring individual performance in an extended test battery, MCSA reveals in which domains a given individual performs below or above the control group mean (sub-normal and supra-normal performance, respectively).

Application of MCSA in adults with AS has revealed heterogeneous EF patterns associated with autistic symptomatology (Hill and Bird, 2006), including both sub- and supra-normal performance (Towgood et al., 2009). A recent study tapping EFs (Gonzalez-Gadea et al., 2013) has shown high task-related variability in individuals diagnosed with either AS or attention deficit hyperactivity disorder. Adults with AS have also shown high inter-individual variability in social cognition domains, including ToM (Baez et al., 2012; Gonzalez-Gadea et al., 2013). The only developmental study employing MCSA in autistic children revealed coexisting abnormalities in ToM, EFs, and central coherence theory (Pellicano, 2010). However, this report included only a few children with AS –it primarily evaluated young children with a diagnosis of autism and pervasive developmental disorder-not otherwise specified.

In this paper, we explore the strengths and weaknesses of children with an AS, using both group analysis and MCSA. Specifically, we aim to detect patterns of inter-individual variability within the population through an extended battery including classical neuropsychological tests as well as measures of intelligence, EFs, and ToM.

We expect higher inter-individual variability in performance across children with AS than across controls. In addition, we hypothesize that MCSA will demonstrate varying patterns of cognitive strengths and weaknesses within individuals and that such variation will be absent in group-comparison analysis. Finally, given that FI has been associated with the cognitive profile of AS (Soulieres et al., 2011) and affords substantial contributions to frontal lobe functions (Duncan et al., 1995; Roca et al., 2010, 2012), we expect that individual differences in FI will partially influence the cognitive profile of AS children.

## Materials and methods

### Participants

Nineteen children with AS and 19 control individuals participated in this study. Individuals in the AS group were selected from the outpatient population of the Institute of Cognitive Neurology (INECO) and were assessed by a psychiatrist. Their diagnosis was based on the criteria established by Diagnostic and Statistical Manual of Mental Disorders (DSM-IV) (American Psychiatric Association, 2000). Additionally, the patients' symptom presentation was measured using the Autism Quotient (AQ) for children (Baron-Cohen et al., 2001b) and adolescents (Baron-Cohen et al., 2006). This questionnaire includes traits of autistic patients which are overlooked in other diagnostic tools (Baron-Cohen et al., 2001b, 2006; Auyeung et al., 2008). We also employed the Social Communication Questionnaire (SCQ; Bolte et al., 2008), which is based on the Autism Diagnostic Interview-Revised, and is widely used in clinical research and practice (Chandler et al., 2007; Norris and Lecavalier, 2010). A psychiatrist then validated the symptom examples provided by the AQ and SCQ and checked the other AS symptoms and criteria.

Twenty-two typically developing children were recruited from neighboring schools. Nineteen of these participants were selected to form a control group, matched for age, gender, and fluid/crystallized intelligence with respect to the AS group. Note that both measures of intelligence were used as matching criteria, so as to prevent the underestimation of intelligence by employing a single criterion (Hayashi et al., 2008; Soulieres et al., 2011). Moreover, given that AS children obtained high variability in fluid intelligence and low variance in crystallized intelligence (see SDs in Table 1), control-participant selection was based on group-wise rather than pair-wise matching criteria. The groups showed no significant differences on any of the matching measures (see Table 1). The following exclusion criteria for both groups were applied: (1) participants who met DSM-IV criteria for any axis-I; and (2) individuals with a history of intellectual disability, neurological disease, psychiatric disease (except AS in patient group), or any clinical condition that may affect cognitive performance.

**Table 1:** **Mean (SD) and range values for baseline characteristics of the participants**.

	**AS (*n* = 19)**	**Controls (*n* = 19)**	p^*^
Age	11.89 (2.64)	10.89 (2.30)	0.222
Range	8–15	8–15	
Gender (Males:Females)	18:1	15:4	0.170
Fluid intelligence	35.70 (13.78)	35.10 (5.76)	0.863
Range	12–57	26–45	
Crystallized intelligence	101.93 (11.96)	100.59 (12.4)	0.763
Range	75–116	85–119	
AQ			
<12 years	84.75 (34.13)	n.a	–
>12 years	30.12 (9.81)	n.a	–
SCQ	19.25 (4.79)	n.a	–

* *Two-tailed student's t Test, except for gender, which as analyzed through the Fisher's Exact Test*.

^*^AQ: Autism Quotient scale (clinical cut-off score of 76 on scale of children under 12 years old and 29 points on scale of adolescents over 12 years old). SCQ: Social Communication Questionnaire (clinical cut-off score of 15).

Parental written informed consent was obtained in accordance with the declaration of Helsinki. The study was approved by the ethics committee of the INECO.

### Neuropsychological assessment

An extended battery of neuropsychological tests was used to assess cognitive functioning, including measures of intelligence, motor speed, memory, visuo-spatial constructional ability, EFs, and ToM.

#### Intelligence

FI was evaluated through the Raven's Progressive Matrices Task RPMT (Raven et al., 1992). We employed the Raven's colored progressive matrices (RCPM) version for children below age 10 and the standard version (RPSM) for the remaining participants. We used standardized norms to convert RCPM scores to RPSM index (Raven, 2008). In addition, the Peabody Picture Vocabulary Test (PPVT, Dunn and Dunn, 1981) was applied to assess crystallized intelligence (CI).

#### EFs

Attention, inhibition, and cognitive flexibility were evaluated using the Stroop task (Spreen et al., 2006) and the Trail Making Test (TMT; Spreen and Gaddes, 1969). To assess response inhibition, we used the Stroop test's index of interference and the number of correct words from the color-word list. Attention and speed processing were evaluated with the TMT-A, and cognitive flexibility through the TMT-B. Furthermore, we considered an interference index (TMT-B minus TMT-A, Bowie and Harvey, 2006). Finally, working memory was assessed using the digit span and arithmetic subtests from the Weschler Intelligence Scale III (WISC III, Wechsler, 1991).

#### ToM

To assess ToM, we applied the RMET (Baron-Cohen et al., 2001c), which consists of 28 photographs of the ocular region of different faces. Participants must select the adjective (in a group of four) that best describes the thoughts or feelings of the individual faces.

#### General neuropsychology

We employed sub-tests from the WISC III (Wechsler, 1991) to evaluate motor processing speed (subtests of coding and symbol search) and expressive vocabulary (vocabulary subtest). In order to test information processing styles, we used a list of unstructured words from the Rey Auditory Verbal Learning Test (RAVLT, Spreen et al., 2006), which evaluates verbal memory acquisition/learning (we included scores for immediate recall, delayed recall, and interference). Additionally, we evaluated immediate and delayed logical memory through the story memory subtest from the Wide Range Assessment of Memory and Learning (WRAML, Adam and Sheslow, 1990). Finally, to assess visuo-spatial constructional ability, we used the copy and visual delayed memory trials of the RCFT (Rey, 1959).

### Data analysis

Group differences were analyzed through an ANCOVA test using age as a covariate. Eta squared (*n*^2^) was employed as a measure of effect size for the significant effects. In addition, we included an inferential test used to assess variance equality between two groups (only significant differences were reported). To further assess inter-individual differences, we conducted MCSA and compared each participant with the mean of the control group on every measure. We followed the method of Towgood et al. (2009) by using a threshold of two standard deviations (SDs) from the mean of the control group to define the normal range. First, we removed control children who displayed extreme performance in each sub-measure, according to the two SD criteria. Second, we recomputed the control means and SDs excluding these subjects and identified AS and control participants whose performance was sub-normal (two SDs below control mean), supra-normal (two SDs above control mean), and average (between −1.99 and 1.99 SDs from the control mean). Third, the participants previously excluded were re-included for MCSA (see **Figures 2A,B**).

We then used non-parametric (Kruskal-Wallis and Mann-Whitney) tests to assess whether the number of measures in which AS individuals obtained sub- and supra-normal performance was associated with individual differences in FI. Finally, we used Spearman's rank correlations to examine the association between FI and neuropsychological measures. The significance of all correlations was corrected for multiple comparisons using the Sidak method. The adjusted α level after correction was set at 0.002. The α value for all other statistical tests (not related to correlation) was set at 0.05.

## Results

### Group differences analyses

**Figure 1:**
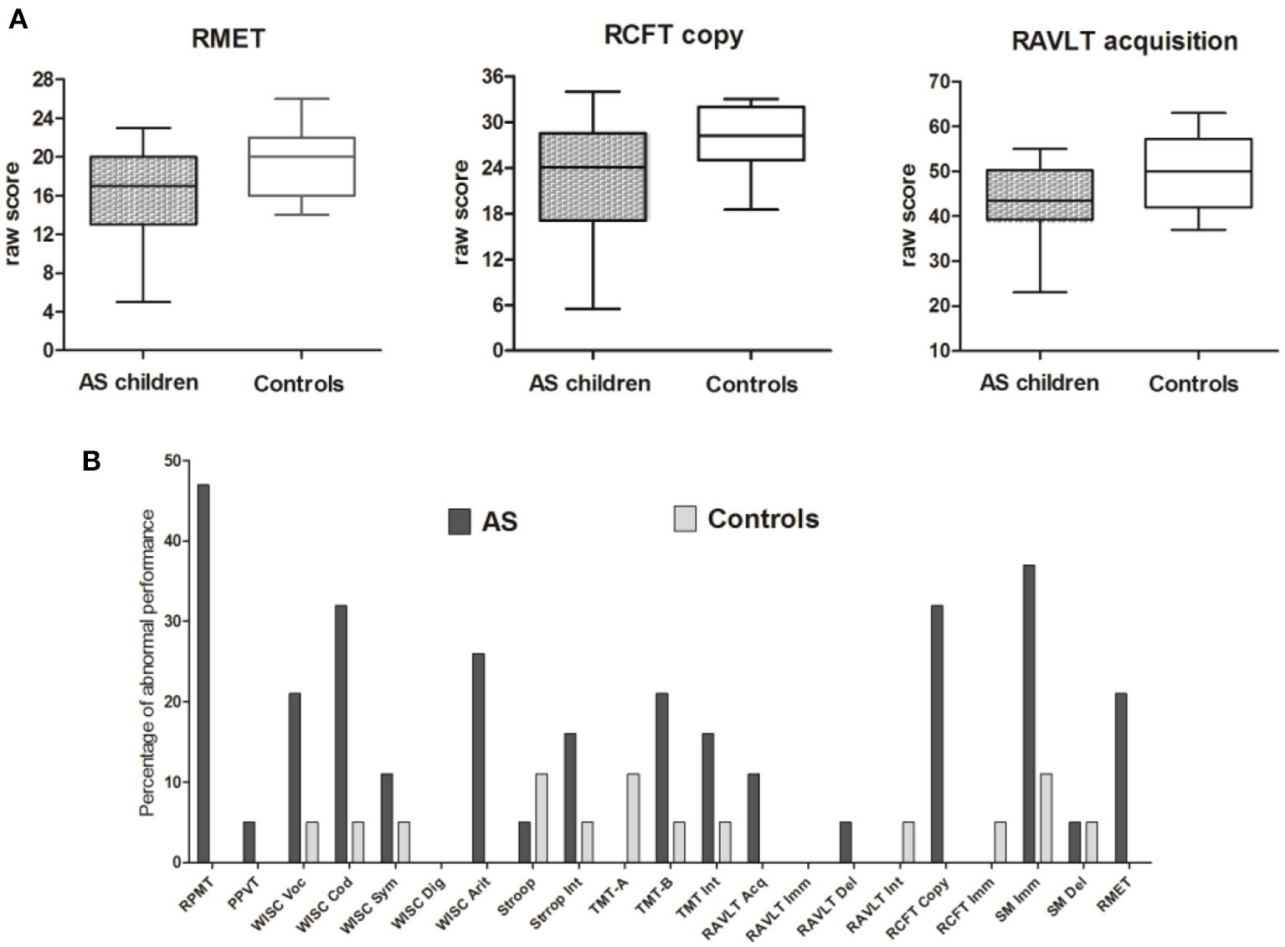
**Summary of main results for group and individual comparisons. (A)** Box plots of each significant group differences between AS and control groups. **(B)** Percentage of individuals with extreme performance on each measure from the neuropsychological assessment. Gray (control group) and black (AS group) columns represent the percentage of individuals with extreme performance (either sub- or supra-normal performance) (see Figures 2A,B for a detailed description). ^*^RPMT, Raven's Progressive Matrices Task; PPVT, Peabody Picture Vocabulary Test; WISC voc, Wechsler Intelligence Scale for Children vocabulary subtest; WISC cod, Wechsler Intelligence Scale for Children coding subtest; Wechsler Intelligence Scale for Children symbol search subtest; Wechsler Intelligence Scale for Children digit span subtest; Wechsler Intelligence Scale for Children arithmetic subtest; StroopInt, Stroop test, interference score; TMT-A, Trail-Making Test part A; TMT-B, Trail-Making Test part B; TMT-Int, Trail-Making Test interference score; RAVLT Acq, Rey Auditory Verbal Learning Test Acquisition; RAVLT Imm, Rey Auditory Verbal Learning Test immediate recall; RAVLT delay, Rey Auditory Verbal Learning Test delay recall; RAVLT Int, Rey Auditory Verbal Learning Test interference score; RCFT copy, Rey Complex Figure Test copy score; RCFT imm, Rey Complex Figure Test immediate recall score; SM Imm, story memory immediate recall; SM delay, story memory delay recall; RMET, Reading-the-mind-in-the-Eyes Task.

Table 2 shows the significance of group comparisons after ANCOVA, using age as a covariate. Following covariation, the AS group was significantly impaired on verbal memory acquisition [RAVLT Acquisition: *F*_(1, 35)_ = 5.72, *p* = 0.024, *n*^2^ = 0.175], visuo-spatial constructional ability [RCFT copy: *F*_(1, 35)_ = 16.60, *p* = 0.000, *n*^2^ = 0.335], and ToM [RMET: *F*_(1, 36)_ = 6.45, *p* = 0.016, *n*^2^ = 0.164] (see Figure 1A). For these differences, age was significantly related to the RCFT [*F*_(1, 35)_ = 17.34, *p* = 0.000, *n*^2^ = 0.344], but not to RAVLT acquisition [*F*_(1, 35)_ = 1.22, *p* = 0.278, *n*^2^ = 0.043] or RMET [*F*_(1, 35)_ = 0.277, *p* = 0.602, *n*^2^ = 0.008].

**Table 2:** **Mean, SDs, and group differences between AS and controls**.

	**AS individuals**	**Controls**	AS vs. Controls^*^		**Correlations with FI**			
	**Mean (*SD*)**	**Mean (*SD*)**	**Group**	**Age**	**AS patients**		**Controls**	
					***r_s_***	***p***	***r_s_***	***p***
Vocabulary WISC III	33.53 (8.42)	36.06 (5.42)	0.105	0.012	0.44	0.079	−0.06	0.802
Arithmetic WISC III	16.84 (2.93)	16.94 (1.56)	0.636	0.103	0.60	0.011	0.52	0.021
Coding WISC III	19.84 (7.86)	21.29 (5.12)	0.286	0.079	0.83	0.000	0.05	0.853
Symbol search WISC III	35.84 (13.98)	40.94 (12)	0.058	0.005	0.73	0.001	0.14	0.569
Digit span WISC III	13.21 (2.95)	14.94 (3.71)	0.088	0.338	0.20	0.453	−0.20	0.459
Stroop	28.81 (9.91)	32.38 (8.72)	0.141	0.036	0.51	0.035	−0.20	0.447
Stroop interference	20.31 (6.84)	18.53 (7.55)	0.798	0.128	0.21	0.441	−0.06	0.821
TMT-A	29.44 (16.06)	27.05 (14.92)	0.631	0.885	−0.17	0.717	−0.32	0.181
TMT-B	86.22 (53.59)	72.89 (26.99)	0.485	0.374	−0.72	0.002	−0.05	0.818
TMT interference	56.77 (42.93)	45.84 (23.1)	0.519	0.245	−0.80	0.000	0.08	0.742
RAVLT acquisition	42.75 (9.46)	49.44 (7.74)	0.024	0.278	0.14	0.667	0.05	0.831
RAVLT immediate recall	10 (2.72)	9.44 (4.16)	0.726	0.741	−0.10	0.741	0.07	0.759
RAVLT delayed recall	9.64 (3.2)	10.63 (2.84)	0.381	0.824	0.39	0.226	0.23	0.358
RAVLT interference	1.27 (1.90)	2.61 (2.66)	0.156	0.712	0.41	0.212	−0.37	0.126
RCFT copy	22.41 (7.59)	27.5 (4.67)	0.000	0.000	0.24	0.372	0.16	0.516
RCFT immediate recall	12.14 (7.41)	15.00 (6.10)	0.104	0.063	0.21	0.440	0.27	0.282
Story memory immediate	18.56 (8.86)	21.2 (4.34)	0.189	0.284	0.40	0.111	0.22	0.353
Story memory delayed	17.94 (9.37)	17.13 (6.25)	0.847	0.860	0.40	0.123	0.14	0.573
RMET	15.94 (5.48)	19.87 (3.6)	0.016	0.602	0.17	0.537	−0.08	0.730

* *p-values of the ANCOVA test for group comparison with age as a covariate. WISC III, Wechsler Intelligence Scale for Children (third version); TMT-A, Trail Making Test part A; TMT-B, Trail Making Test part B; RAVLT, Rey Auditory Verbal Learning Test; RCFT, Rey Complex Figure Test; RMET, Reading-the-Mind–in-the-Eyes Test*.

Although participants with AS presented lower scores on receptive vocabulary (vocabulary from WISC III), working memory (digit span and arithmetic from WISC III), motor processing speed (coding and symbol search from WISC III), cognitive flexibility (TMT-B), response inhibition (Stroop task), and logical memory (story memory from WRAML), no significant differences were observed in these measures. However, the SDs of some measures were higher in the AS group (see Table 2). A test comparing the group variance revealed significantly higher SDs for the AS than control participants in the RPMT (*p* = 0.000), RCFT copy (*p* = 0.016), and story memory immediate recall (*p* = 0.001). Consistent with previous reports (Hill and Bird, 2006; Towgood et al., 2009; Baez et al., 2012; Gonzalez-Gadea et al., 2013), these data show a consistent pattern of high inter-individual performance variability in AS.

### Multiple case series analyses (MCSA)

**Figure 2:**
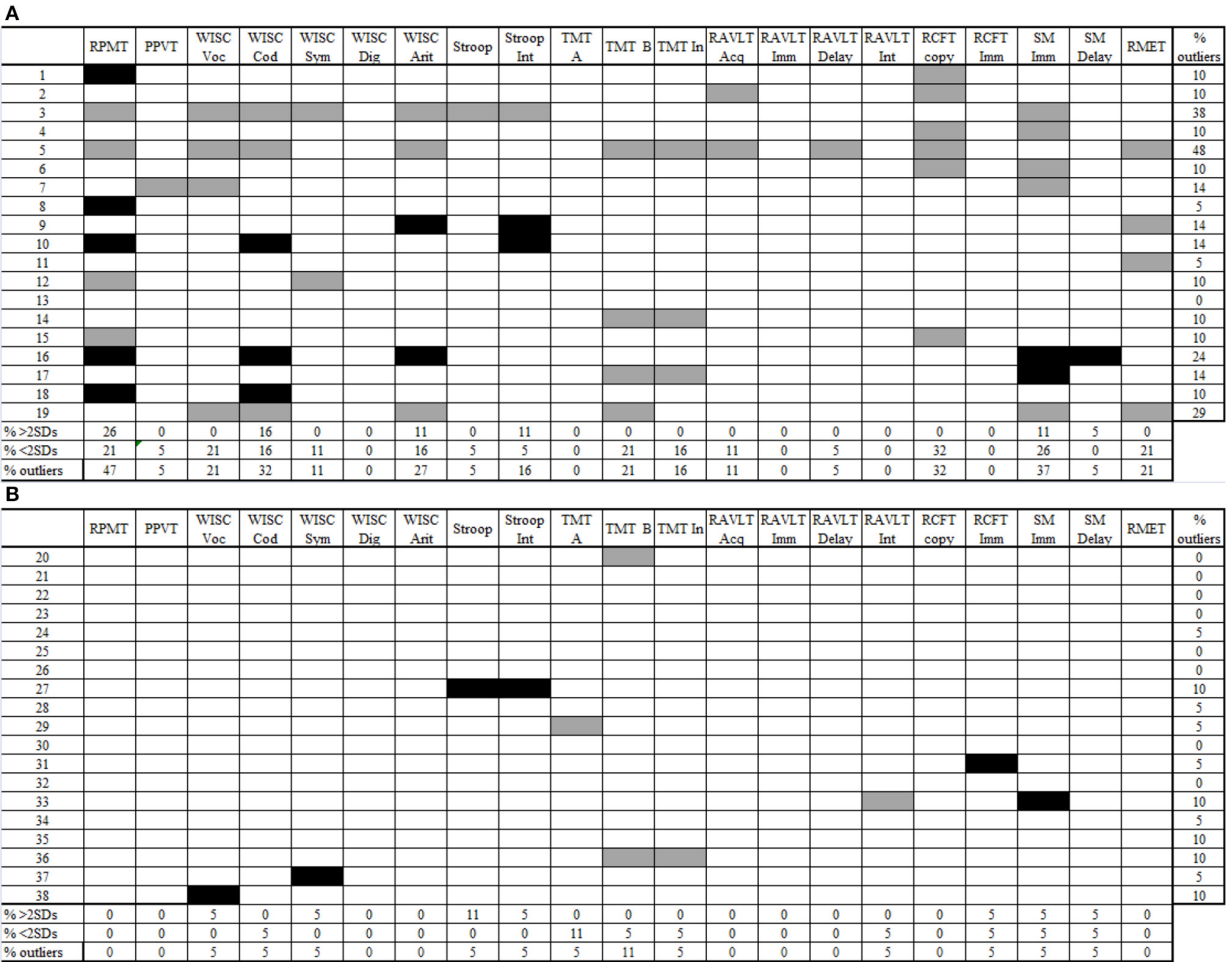
**Individual profiles of performance for AS children (2A) and controls (2B)**. Gray cells show performance 2 SDs below the control mean (sub-normal). Black cells show performance 2 SDs above the control mean (supra-normal). Blank cells show performance between −1.99 and 1.99 SDs according to the control mean (normal performance). RPMT, Raven's Progressive Matrices Task; PPVT, Peabody Picture Vocabulary Test; WISC voc, Wechsler Intelligence Scale for Children vocabulary subtest; WISC cod, Wechsler Intelligence Scale for Children coding subtest; Wechsler Intelligence Scale for Children symbol search subtest; Wechsler Intelligence Scale for Children digit span subtest; Wechsler Intelligence Scale for Children arithmetic subtest; Stroop Int, Stroop test, interference score; TMT-A, Trail-Making Test part A; TMT-B, Trail-Making Test part B; TMT-B, Trail-Making Test interference score; RAVLT Acq, Rey Auditory Verbal Learning Test acquisition; RAVLT Imm, Rey Auditory Verbal Learning Test immediate recall; RAVLT delay, Rey Auditory Verbal Learning Test delay recall; RAVLT Int, Rey Auditory Verbal Learning Test interference score; RCFT copy, Rey Complex Figure Test copy score; RCFT imm, Rey Complex Figure Test immediate recall score; SM Imm, story memory immediate recall; SM delay, story memory delay recall; RMET, Reading-the-Mind-in-the-Eyes Task.

First, we explored performance variability among AS children (Towgood et al., 2009). For each group, in every measure, we calculated the percentage of sub- and supra-normal performers and the percentage of outliers (sub-normal plus supra-normal performance). In the control group, the maximum percentage of outliers was 11% (see Figure 2B). Regarding the AS group, 10 out of 21 measures exceeded this maximum percentage.

In this group, some measures revealed only sub-normal performance: the copy of RCFT, with 32% of sub-normal performers; and TMT-B, vocabulary from WISC-III, and RMET, with 21% sub-normal performers. However, the highest proportion of outliers was observed in tasks where individuals obtained both sub- and supra-normal performance. Thus, the RPMT exhibited the highest proportion of participants with extreme performance (47%), followed by SM immediate recall (37%), coding from WISC III (32%), and arithmetic (27%). There were no participants who performed only supra-normally.

Therefore, we used the Kruskal-Wallis test to compare the number of measures with sub- and supra-normal performance according to the patients' FI score. Following Towgood et al. (2009), we separately recounted the number of measures (except FI) in which each AS individual obtained sub and supra-normal performance. Additionally, we used FI to categorize three groups of participants with inferior (<2 SDs), superior (>2 SDs), and average (between −1.99 and 1.99 SDs) scores. Table 3 shows that significant group differences were observed only in the number of measures in which participants obtained sub-normal performance (*H* = 8.37, *p* = 0.015). After that, we used Mann-Whitney tests for pair-wise comparisons. Participants with superior FI scores displayed a smaller number of measures with sub-normal performance than children with inferior (*U* = 1.00, *p* = 0.019) and average (*U* = 4.00, *p* = 0.007) FI scores. No significant differences between AS children with average and inferior FI (*U* = 16.00, *p* = 0.558) were observed. These results suggest that AS children with higher FI have a lower probability of showing deficiencies in other domains.

**Table 3:** Extreme ranges of performance of AS participants divided in terms of individual differences in FI^*^.

	**Average FI**	**Inferior FI**	**Superior FI**	***p*^a^**
	***N* = 10**	***N* = 4**	***N* = 5**	
Sub-normal performance				0.015
Median	2	4	0	
Range	0–6	1–9	0–1	
Supra-normal performance				0.099
Median	0	n.a	1	
Range	0–2	n.a	0–4	

* *Number of measures from the neuropsychological battery (except RPM) where performance was either 2SDs below (sub-normal performance) or above (supra-normal performance) from the control mean (see Section Multiple Case Series Analyses (MCSA) for description of process to divide FI groups)*.

a *^a^ Kruskal-Wallis test*.

### Relationship between FI and other cognitive domains

Finally, to further explore the influence of FI on other domains, we conducted a correlation analysis between FI (RPMT) and neuropsychological measures in each group (see Table 2).

**Figure 3:**
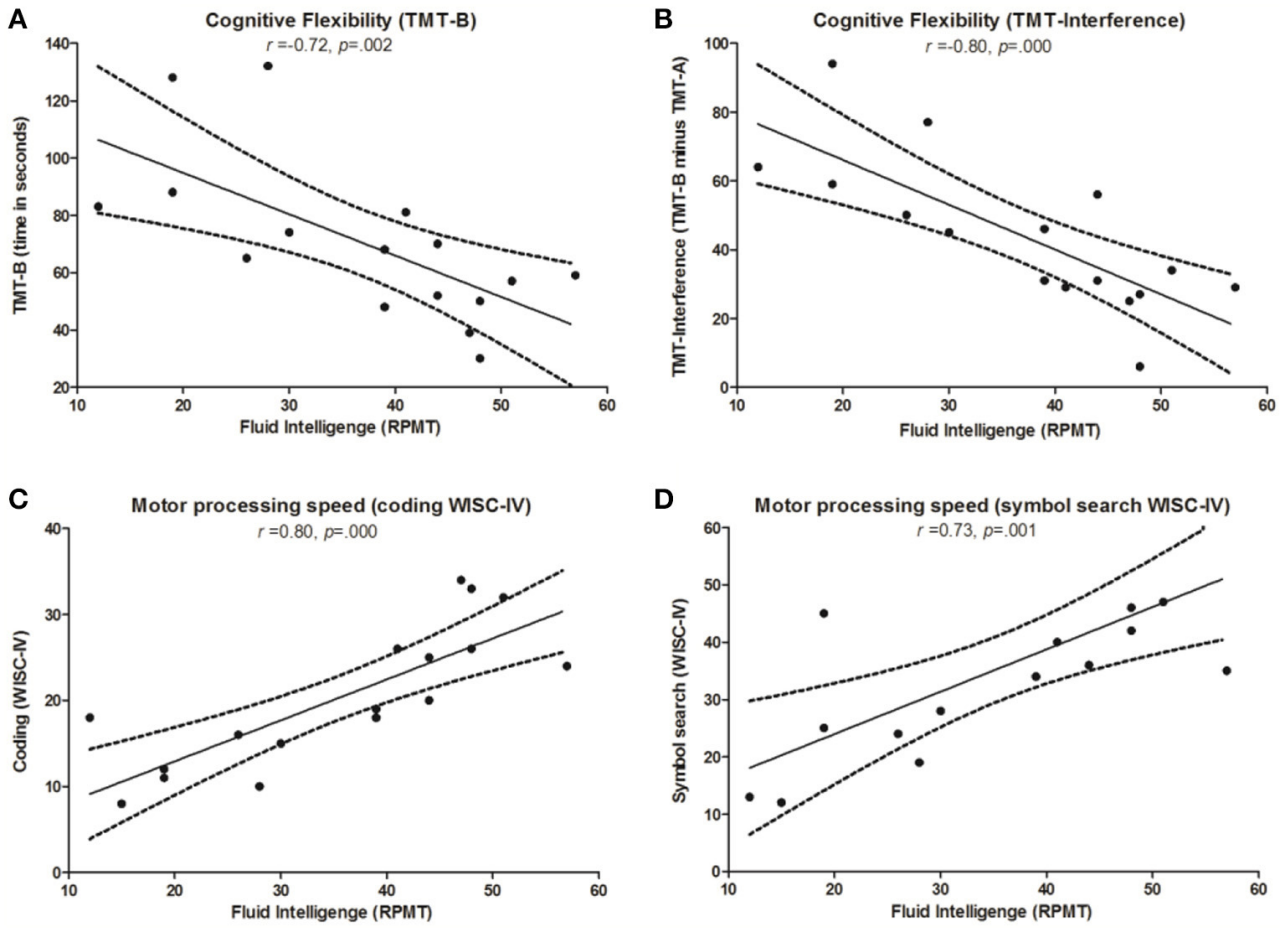
**Correlations between FI and cognitive tasks in AS children**. Significant correlations in the AS group between Raven's Progressive Matrices Task (RPMT) and: **(A)** Trail-Making Test part B (TMT-B); **(B)** Trail-Making Test, interference score (TMT-Int); **(C)** Coding subtest from the Wechsler Intelligence Scale for Children (coding WISC-IV); **(D)** symbol search subtest from Wechsler Intelligence Scale for children (symbol search WISC-IV).

For the AS group, there was a significant association between FI and cognitive flexibility (TMT-B: *r_s_* = −0.72, *p* = 0.002; TMT Interference: *r*_s_ = −0.80, *p* = 0.000). A significant correlation was also found between FI and motor processing speed (coding: *r_s_* = 0.83, *p* = 0.000; symbol search: *r_s_* = 0.73, *p* = 0.001) (see Figure 3). For the control group, we found no significant correlation between FI and neuropsychological tasks.

In summary, MCSA revealed that group comparison analyses are blind to the heterogeneity in children with AS. Furthermore, AS individuals with higher scores on FI evidenced fewer difficulties in other cognitive domains and improved performance in cognitive flexibility and processing speed tasks.

## Discussion

The present study assessed the heterogeneity of children with AS during cognitive tasks and the commonalities associated with this variability. In addition to their well-known difficulties in ToM and global information processing, the AS group showed high inter-individual variability (sub- and supra-normal performance) across cognitive tasks. At the individual level, higher FI was associated with less cognitive difficulties and high cognitive flexibility and motor processing speed. To our knowledge, this is the first application of MCSA in children with AS and the first report of partial influence of FI on the cognitive profile of these patients.

### Abnormalities in ToM and information processing

The results demonstrated significant group differences between AS and control children in RMET. This suggests that AS children have difficulties in inferring the mental states of others. However, the MCSA showed that only a few children of the AS sample performed sub-normally, confirming that group differences are not always a reliable index of deficits in the AS group. Furthermore, as in the case of AS adults (Ponnet et al., 2004; Spek et al., 2010; Baez et al., 2012; Gonzalez-Gadea et al., 2013), the RMET might not be a sensitive instrument for detecting ToM abnormalities in AS children.

In line with previous evidence of global processing deficits in autism (Minshew and Goldstein, 1998), our AS group showed failures in visuo-spatial constructional abilities (RCFT) and memory/learning acquisition (RALVT). Other studies using visuo-spatial tasks have shown that individuals with AS tend to focus on details rather than global figures, a strategy that is ineffective for RCFT performance (Prior and Hoffmann, 1990; Mottron and Belleville, 1993; Le Sourn-Bissaoui et al., 2011). Since this task requires information encoding supported by organizing and planning strategies (Watanabe et al., 2005; Ogino et al., 2009), difficulties in EFs may underlie these results. In the same vein, when participants are instructed to learn a list of words, inefficient encoding strategies would imply lower acquisition and recall of information (Minshew and Goldstein, 2001; Bowler et al., 2008). Patients with EF impairment perform poorly on unstructured word-list memory tasks, but not on logical memory tests (Tremont et al., 2000; Brooks et al., 2006; Torralva et al., 2011). Our AS participants exhibited difficulties in RAVLT acquisition but not in logical memory tests (story memory). The discrepancy between the memory tests may reflect executive difficulties in organizing efficient information encoding strategies. However, MCSA revealed that six children with AS exhibited inferior performance in the RCFT, while only two children obtained sub-normal scores in the RAVLT. This suggests that difficulties in global processing tasks are also heterogeneous between AS children.

In addition, as was the case in previous studies (Ambery et al., 2006; Hill and Bird, 2006; Nyden et al., 2010), we found no significant differences between groups on the EF tasks However, we did find marked inter-individual variability in these functions, which may account for the absence of group differences. In this sense, Liss et al. (2001) suggested that the problem of universality in executive dysfunction in ASD is that most studies focus on group differences, neglecting individual variations. Our results support this view.

### Inter-individual variability among AS individuals

Various authors agree that research on AS should abandon the search for a single cause and address it as a complex, multifactorial syndrome (Happe et al., 2006b; Willcutt et al., 2008; Brunsdon and Happe, 2014). In our study, performance on cognitive measures was more heterogeneous in AS than control children (see Figure 1B). The patients showed extreme performance, including sub-normal (<2 SD), supra-normal (>2 SD), and combined scores. Consistent with our findings, previous reports showed similar patterns among adults with AS (Towgood et al., 2009; Baez et al., 2012; Gonzalez-Gadea et al., 2013). Ours is the first study to confirm this cognitive profile in children with AS.

MCSA of the AS group revealed that several patients presented sub-normal performance in domains that may be associated with their diagnostic categories, such as social interaction (ToM), verbal communication (receptive vocabulary), and repetitive interests and activities (cognitive flexibility). Moreover, the group exhibited both sub- and supra-normal performance in domains excluded from diagnostic criteria, such as information processing styles (logical memory test and RCFT), processing speed (coding from WISC III), and working memory (arithmetic from WISC III).

In support of our first hypothesis, subtle differences across cognitive domains were not revealed by the group-type analysis. Thus, in domains associated with the AS diagnosis, this group included a high proportion of individuals with sub-normal performance, while in other domains, AS children obtained either sub- or supra-normal performance.

For instance, although AS and control groups were similar regarding FI, the former showed high inter-individual variability on the RPMT. Indeed, this group displayed the greatest variability in FI. Most previous reports described FI as either an intact or superior ability in individuals with AS (Hayashi et al., 2008; Chen et al., 2010; Morsanyi and Holyoak, 2010; Soulieres et al., 2011). However, these studies failed to mention the patients' strong variability in the RPMT. For example, in the study by Soulieres et al. (2011), the SDs of the AS group on this task were more than two times larger than in the control group.

Since FI may be understood as a general intelligence factor that contributes to all cognitive functions (Spearman, 1904), we hypothesized that individual differences in FI should affect the cognitive profile of AS participants. We found significant differences between participants with high, low, or average FI in terms of the number of cognitive measures with sub-normal performance. Participants with superior FI demonstrated less impairment in other cognitive functions. Similarly, most executive deficits in patients with frontal dysfunctions are explained by a loss in FI (Duncan et al., 1995; Roca et al., 2010; Woolgar et al., 2010). Likewise, improved FI performance is related to better psychosocial adaptation in typically developing children (Huepe et al., 2011). Our results suggest that high FI may reduce AS children's vulnerability to develop deficits in other cognitive functions.

### Relationship between FI and specific cognitive domains

In view of previous findings, we investigated the association between FI and other cognitive functions in both AS and control groups. Correlation analyses revealed that FI was linked to cognitive flexibility and processing speed only for children with AS. Our data are consistent with recent studies suggesting that FI is a substantial contributor to classical EF tasks (such as TMT) in neurological patients (Roca et al., 2010, 2012). However, this effect cannot be attributed to a positive correlation between FI and all frontal functions. In the present study, ToM was not correlated with FI, as reported elsewhere in the literature (Roca et al., 2010, 2012).

Previous reports indirectly support our findings. First, Kaland et al. (2008a) suggested a link between low FI and limited cognitive skills to solve visuo-constructional problems in a group of children with AS. Second, better attention switching has been shown to predict a higher RPMT overall score in typically developing individuals with variants of the autistic phenotype (Fugard et al., 2011). Finally, Soulieres et al. (2011) suggested that high FI in children with AS would provide them with better mechanisms to solve cognitive tasks.

Finally, the relationship between FI and EFs has been supported by functional magnetic resonance imaging (fMRI) studies. The lateral prefrontal cortex and posterior parietal regions are the neural substrates subserving the relation between abstract reasoning (FI) and performance on EF tasks among neurotypical adults (Gray et al., 2003; Lee et al., 2006; Woolgar et al., 2010). For their own part, participants with AS exhibit significantly increased activation in the lateral prefrontal cortex and left parietal brain regions during EF tasks (Schmitz et al., 2006). Increased brain activation has been explained by abnormal brain anatomy or the use of alternative cognitive strategies (but see opposed results in autism: Luna et al., 2002; Koshino et al., 2005; Soulieres et al., 2009).

By combining evidence from MCSA and correlation analysis, we offer preliminary evidence that AS children with strengths in FI may develop efficient strategies to perform some cognitive tasks. However, the neural mechanisms underlying FI and cognitive functioning in individuals with an AS diagnosis remain unknown. Futures studies should investigate the neural networks associated with this interaction.

### Limitations and further directions

The present data confirm heterogeneous cognitive profiles in classical neuropsychological tests among children with AS. However, the origins of this variability remain unknown. Furthermore, previous studies have demonstrated that variation in social cognition tasks is a feature of AS adults (Baez et al., 2012; Gonzalez-Gadea et al., 2013). Future research should explore the issue of heterogeneity in social cognition among AS children.

This study provides preliminary evidence indicating that individual differences in FI may be associated with the heterogeneous profile of strengths and weaknesses of children with AS. Nevertheless, the results should be extended and replicated. The limited sample size in this study did not allow us to establish a definitive causal relationship between FI and heterogeneous cognitive profiles. Regression analyses and structural equation modeling (SEM), which require an extended number of participants, could be robust models to predict the role of FI in cognitive profiles.

Furthermore, the observed heterogeneity may allow for alternative explanations. For instance, clinical measures of autistic symptomatology have been associated with cognitive heterogeneity in adults with AS (Hill and Bird, 2006). Future studies should replicate these findings in children with AS. Finally, traditional approaches interpret variability as *noise* in the data, which was attributed to limitations of the AS diagnosis, leading to multiple subgroups of patients (Towgood et al., 2009). The DSM-5 has incorporated the AS diagnosis within ASD, in an attempt to account for the variations in symptoms and the multifaceted cognitive profile of each patient (Brunsdon and Happe, 2014). Future studies should investigate the sensitivity of this new diagnosis to account for the heterogeneous cognitive profile of these children.

## Conclusions

We suggest that heterogeneity is a defining feature of the cognitive profile of children with AS. These findings have important implications for the treatment, identification, and assessment of these individuals. The DSM-5 has included AS within ASD, which incorporates even more variability in symptoms and behavior among these individuals. The challenge for clinical practice is to work with extensive and flexible neuropsychological assessments that allow for the identification of both deficits and strengths in individuals with AS, so as to hone individual treatment.

The data showed that AS children, as a group, present common difficulties in ToM and global information processing. At the individual level, they demonstrated a wide range of variation in most of the cognitive functions evaluated. Thus, despite extensive research seeking a typical cognitive profile of individuals with AS, the evidence suggests that this syndrome is characterized by an uneven pattern of cognitive strengths and weaknesses. The present report of inter-individual cognitive variability in children with AS aligns with similar findings in autism and adults with AS.

Moreover, a detailed MCSA revealed that individual differences in FI may be associated with this heterogeneous profile. The data showed that high FI was related to fewer cognitive impairments. In addition, FI was associated with cognitive flexibility and motor processing speed only in AS children. The current report is the first to highlight the possible influence of FI on AS cognitive profiles. Indeed, superior abilities in abstract reasoning could compensate or reduce AS children' vulnerability to develop other deficits in cognitive abilities. Further research is needed to elucidate the relationship between FI and the cognitive functioning of these individuals.

### Conflict of interest statement

The authors declare that the research was conducted in the absence of any commercial or financial relationships that could be construed as a potential conflict of interest.
